# Qualitative analysis of programmatic initiatives to text patients with mobile devices in resource-limited health systems

**DOI:** 10.1186/s12911-016-0258-7

**Published:** 2016-02-06

**Authors:** Sachin K. Garg, Courtney R. Lyles, Sara Ackerman, Margaret A. Handley, Dean Schillinger, Gato Gourley, Veenu Aulakh, Urmimala Sarkar

**Affiliations:** 1 Division of General Internal Medicine and Center for Vulnerable Populations at San Francisco General Hospital, University of California, San Francisco (UCSF), San Francisco, USA; 2 Department of Social and Behavior Sciences, UCSF, San Francisco, USA; 3 Center for Care Innovations, Oakland, USA; 4 Department of Epidemiology and Biostatistics, UCSF, San Francisco, USA

**Keywords:** Texting, Mobile technology, Telehealth, Implementation, Qualitative, Safety net, Underserved, PCMH, Consent

## Abstract

**Background:**

Text messaging is an affordable, ubiquitous, and expanding mobile communication technology. However, safety net health systems in the United States that provide more care to uninsured and low-income patients may face additional financial and infrastructural challenges in utilizing this technology. Formative evaluations of texting implementation experiences are limited. We interviewed safety net health systems piloting texting initiatives to study facilitators and barriers to real-world implementation.

**Methods:**

We conducted telephone interviews with various stakeholders who volunteered from each of the eight California-based safety net systems that received external funding to pilot a texting-based program of their choosing to serve a primary care need. We developed a semi-structured interview guide based partly on the Consolidated Framework for Implementation Research (CFIR), which encompasses several domains: the intervention, individuals involved, contextual factors, and implementation process. We inductively and deductively (using CFIR) coded transcripts, and categorized themes into facilitators and barriers.

**Results:**

We performed eight interviews (one interview per pilot site). Five sites had no prior texting experience. Sites applied texting for programs related to medication adherence and monitoring, appointment reminders, care coordination, and health education and promotion. No site texted patient-identifying health information, and most sites manually obtained informed consent from each participating patient. Facilitators of implementation included perceived enthusiasm from patients, staff and management belief that texting is patient-centered, and the early identification of potential barriers through peer collaboration among grantees. Navigating government regulations that protect patient privacy and guide the handling of protected health information emerged as a crucial barrier. A related technical challenge in five sites was the labor-intensive tracking and documenting of texting communications due to an inability to integrate texting platforms with electronic health records.

**Conclusions:**

Despite enthusiasm for the texting programs from the involved individuals and organizations, inadequate data management capabilities and unclear privacy and security regulations for mobile health technology slowed the initial implementation and limited the clinical use of texting in the safety net and scope of pilots. Future implementation work and research should investigate how different texting platform and intervention designs affect efficacy, as well as explore issues that may affect sustainability and the scalability.

**Electronic supplementary material:**

The online version of this article (doi:10.1186/s12911-016-0258-7) contains supplementary material, which is available to authorized users.

## Background

Improving patient-provider communication and the tracking of clinical disease parameters between outpatient medical visits is important for delivering higher-quality primary care under the Patient Centered Medical Home (PCMH) model [1]. As a result, health systems are exploring digital communication solutions, many of which require the Internet or sophisticated health information technology (IT) capabilities [2]. Developing and implementing these solutions may be more challenging for ‘safety net’ health systems, defined by the Institute of Medicine as health systems in the United States (US) that deliver a significant level of healthcare to uninsured, low-income, and other vulnerable patients either by legal mandate or because these patients represent a substantial portion of the patient mix [3]. Core safety net health systems include public hospitals, community health centers, and local health departments. Safety net systems have limited financial resources and less robust IT infrastructure [4], but face the same pressures to expand access and improve their clinical services while also containing costs in the current reform environment [5].

Mobile text messaging (or SMS) may offer an affordable and ubiquitous digital health solution for safety net health systems. The majority of Americans own a cell phone and can send and receive text messages, regardless of their ethnicity, income or education level [6]. Relatively fewer Americans have access to the Internet through a smartphone or home broadband connection, and fewer still in minority and low-income populations [7]. It is not surprising that a majority of safety net patients have expressed a willingness to use text messages to communicate with their healthcare providers [8–10].

Texting has been studied in non-safety net settings for a variety of administrative functions, such as appointment reminders, and clinical functions, such as smoking cessation, glycemic control, and behavioral health management [11–19]. While evidence supporting the clinical effectiveness of texting programs is still emerging, we are not aware of any implementation research to date that has been conducted to formally study the process of designing and adopting these texting programs [20–23], particularly with safety net populations and the systems that disproportionately care for them [24–29]. Safety net systems in particular may face unique barriers (such as financial resource constraints or lagging health IT sophistication) to adoption [30], and formative evaluation of these implementation complexities is needed to explore the promise of texting from an organizational perspective [24].

In order to develop an in-depth understanding of why texting is not more widely used to improve medical services for safety net patient populations, we sought to identify the facilitators and barriers to implementation in real-world safety net settings. Specifically, we interviewed and qualitatively studied a diverse group of large safety net healthcare systems in the state of California that received modest, local extramural funding to pilot their own unique texting programs.

## Methods

We interviewed safety net healthcare systems several months after they received pilot funding through a competitive application process in which they proposed to develop and implement a texting program of their choosing. Ethical approval was obtained from the Institutional Review Board at the University of California, San Francisco. In January 2014, the Center for Care Innovations (CCI; careinnovations.org) awarded eight $30,000 piloting grants (selected from 17 applicants) through their “Texting for Better Care” initiative that was funded by Blue Shield of California Foundation. Eligible applicants were publicly owned and not-for-profit health systems in California that provide comprehensive primary care services to a predominantly underserved patient population [31]. Applicant health systems were required to identify a patient need and target population of at least 75 patients, as well as develop a formal proposal for a texting solution (including the implementation model and plan for sustainability beyond the pilot period) that was scored and voted on using strict assessment criteria. Selected safety net organizations treated an ethnically diverse mix of patients and included many organizational structures, including federally qualified health centers and both privately and publicly funded community health systems (e.g. public hospital systems). CCI provided circumscribed technical assistance for vendor selection and program design. Pilot sites were also given access to informational webinars/seminars on selected topics and invited to voluntarily participate in four ad-hoc “learning exchange calls” to share their implementation experiences with other grantees.

A single telephone interview was conducted with each pilot site and focused on understanding the implementation experiences in order to identify the potential facilitators and barriers encountered during the launch process. Logistical limitations prevented us from performing in-person interviews since the participating sites were geographically located across the entire state of California. Each study author conducted one of the eight telephone interviews corresponding to the eight participating pilot sites. The assignment process for matching an author to a particular study site for interviewing was random. While we recognized the limitations of using multiple interviewers prior to conducting the interviews (discussed later), we felt that each author would be better able to participate in analytic discussions if they were actively engaged in the data collection process. The impetus for focusing our interviews on the implementation process stems from the recently created “Public Healthcare system Evidence Network and Innovation eXchange” (PHoENIX). This exchange is tasked with sharing best practices and applying implementation science methods to investigate how innovations are employed and sustained by public healthcare systems throughout California. PHoENIX is comprised of our team of practice-based researchers and the California Association for Public Hospitals’ Safety Net Institute, which represents all 21 public hospital systems in California.

In order to initiate these interviews, CCI emailed and obtained informed consent from the texting pilot project leads and involved team members from all eight participating health systems, inviting them to voluntarily interview with our research team in October 2014. At least one member from each site volunteered to be interviewed by our team, although four of the eight sites had multiple volunteers that participated in the site interview. The backgrounds and roles of volunteer participants across all of the eight sites ranged from leadership (such as pilot program managers) to texting platform vendor partners and frontline project staff (nurses, physicians, health coaches, clerical, and IT). To develop contextually relevant questions for each site, we drew from quarterly progress reports (at three- and six-month time points) submitted to CCI by an independent evaluation group (see acknowledgements) contracted to assess the texting pilots and also provide periodic and direct technical support to the grantees. Our implementation science-focused project, however, was separately and independently performed from the evaluative assessment requested by CCI from this independent evaluation group.

### Interview process and instrument

**Fig. 1:**
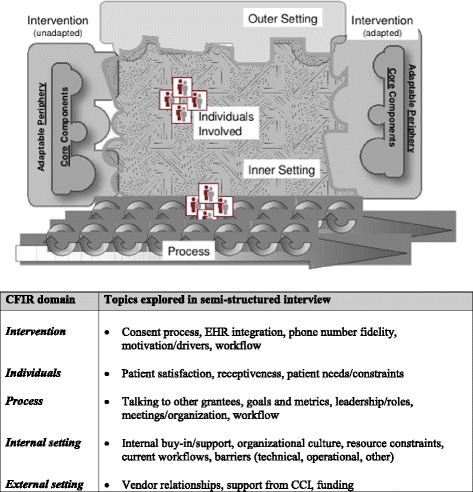
Interview topics explored using the Consolidated Framework for Implementation Research (CFIR)

We developed a semi-structured interview guide (Additional file 1) using the Consolidated Framework for Implementation Research (CFIR) as our primary guide [32]. CFIR is a widely used set of constructs in implementation science that we used as a conceptual guide for systematically assessing the implementation of an innovation. CFIR facilitates formative evaluations through the assessment of five major analytic domains that are thought to influence implementation: 1) intervention characteristics (e.g., adaptability, strength of the evidence, cost, or relative advantage), 2) implementation process (e.g., planning, engaging, executing, or evaluating), 3) characteristics of the individuals involved (e.g., self-efficacy, knowledge and beliefs about the intervention), 4) internal contextual factors (e.g., culture, readiness for implementation and change, or structural architecture of the organization), and 5) external contextual factors (e.g., external policies or mandates, guidelines, or peer pressure). While we anchored the general topics explored in interview questions to this conceptual framework (Fig. 1), the use of a semi-structured interview schedule allowed for additional themes (separate from CFIR) to emerge from participant responses as well. We performed a one hour-long interview with each of the eight pilot sites, and a different member of our research team conducted each telephone interview. Interviews were audio recorded and professionally transcribed, but we were unable to accurately ascribe responses to each interviewee. To protect participant confidentiality, we have not provided raw interview transcripts.

### Data analysis

All authors participated in the data analysis, which took place in several stages. First, the entire research team reviewed the interview transcripts and conducted a broad discussion of themes. Second, two authors (SG, US) read all the transcripts in detail and subsequently met to reach agreement on a comprehensive list of potential themes related to commonly reported implementation facilitators and barriers, using both inductive (identification of themes) and deductive (based on CFIR domains) approaches. Third, two authors (SG, US) manually identified representative quotes for each theme. We recorded and organized the identified themes and quotations in Microsoft Word and Excel without using coding software due to the modest sample size and transcript length. Fourth, all authors collectively met to discuss the extracted themes and focus the analysis on selected critical themes based on how commonly they were reported by pilot sites, relative importance given the existing literature, and perceived validity or utility by methodological and content experts on the team. Fifth, two authors (SG, US) re-read the transcripts and agreed upon a revised list of themes based on input from the other authors. Sixth, SG identified and extracted relevant site-specific characteristics and statistics (Table 1) from interview data such as the roles of interview participants (if available from transcripts) or whether or not pilot sites had prior texting experience. SG used a Microsoft Excel spreadsheet to record this additional information, which was then reviewed by a second author (US). Last, all authors iteratively revised and agreed upon the final list of themes and representative quotes, as well as their description in the final manuscript. Consistent with our goals, we broadly categorized themes into facilitators or barriers of the implementation process. Conflicts were resolved through discussion by the authors. Information from all eight interviews contributed to the final analysis.

**Table 1:** Pilot site information and research context *

Site ID	Interviewee Roles (number)	Prior texting experience	Launch phase	Platform directionality	Consent process	PHI in text content	EHR integration
A	• Community health advocate and texting project manager (1)	Yes	Pre-	Unidirectional	Opt-in	No	No
B	• EHR programmer (1) • Strategic project manager (1) • Operations manager (1)	No	Post-	Bidirectional	Opt-out	No	Yes
C	• Physician project lead (1)	No	Pre-	Bidirectional	Opt-out	No	No
D	• Director of Public Health Programs, Chronic Disease and Health Education (1)	Yes	Post-	Bidirectional	Opt-in	No	Yes
E	• Clerical front office staff (1) • Referral specialist (1) • Project director (1)	No	Post-	Unidirectional	Opt-in	No	Yes
F	• Physician and Department Chief (1) • Physician lead of health coaching program (1) • Vendor representative (1) • Site Project Manager (1)	No	Post-	Bidirectional	Opt-in	No	No
G	• Project Director (1)	Yes	Post-	Unidirectional	Opt-in	No	No
H	• Medical Director of Quality Improvement Community programs (1) • Medical Director of Care Coordination (1) • Research coordinator (1)	No	Post-	Unidirectional	Opt-in	No	No

* Telephone interviews were transcribed using professional software. Platform directionality refers to the ability for the health system to message patients and vice versa. Unidirectional platforms do not allow patients to message the health system. “Opt-in” consent processes require each patient to be consented before enrollment without assuming consent initially (which is an “opt-out process). EHR integration involves automating the interface of information from the texting platform with the EHR of the health system. Abbreviations include: *ID* identification code, *PHI* Protected health information (patient-identifying). *EHR* Electronic health record

## Results

All safety net health systems (*n* = 8) completed an hour-long interview with research study staff, representing 17 volunteer interviewees across all the sites with a wide variety of roles in the organization (Table 1). Every site reported having partnered with an external vendor to provide the texting platform capability; only three sites had prior experience with implementing a patient texting program (Table 1). At the time interviews were conducted, six sites had already launched their pilots. Two sites were delayed in beginning their pilots: 1) Site C was still waiting for administrative approvals despite a completed platform and design, and 2) Site A was still waiting for their vendor to complete a customized platform. Below we discuss in detail additional contextual factors that are also provided in Table 1 for each texting program and associated pilot site, including platform directionality (unidirectional, bidirectional), consent process (opt-in, opt-out), delivery of protected health information in text message content, and integration of the electronic health record (EHR) with the texting platform. Each site used texting for a different care management purpose, including medication adherence, chronic disease management, behavioral health, patient monitoring, appointment reminders and scheduling, care coordination, and health education and promotion (Table 2). Pilot programs also aimed to optimize patient access, discharge planning and care coordination.

**Table 2:** Clinical uses of texting programs and site information

Current clinical pilot	Pilot Sites	Description of use of texting	Texting goals and metrics	
			Patient-level	System-level
Outreach to uninsured youth	A	To provide insurance coverage information to uninsured youth (ages 18-24)	• Increase health insurance enrollment • Patient satisfaction rate with texting program	• Reduce need for health educators to manually text patients
Appointment reminders	B, H	Appointment reminders for routine outpatient visits and/or hospital discharge appointments	• Improve post-discharge care • Improve patient satisfaction, access and engagement	• Reduce manual calling of patients • Reduce no-show rates • Reduce readmission rates after discharge
Post-discharge care coordination	C	To verify if patients discharged from inpatient or emergency room have follow-up and medications	• Address unmet needs of patients after a “rescue event” (inpatient or ED visit)	• Reduce inpatient and ED readmissions • Decrease costs of capitated contracts
Blood pressure management	D	To communicate about blood pressure with providers for patients in a home-monitoring program	• Improve patient engagement at home • Provide feedback on blood pressure in between visits	• Reduce disparities within patient panels • Reach patients outside clinic with limited access
Specialist referral reminders	E	To provide reminders and information to patients regarding new non-urgent referral appointments	• Provide patient-centered care • Strengthen care coordination in the PCMH model	• Optimize time of the referral managers • Improve PCP-specialist communication • Close open referrals more quickly
Behavioral health - substance abuse recovery	G	Sending motivational messages to patients enrolled in an existing in-person recovery program for substance abuse	• Improve completion rate of 12 week recovery program • Improve patient satisfaction	• Extend communication of the “medical home”

**Fig. 2:**
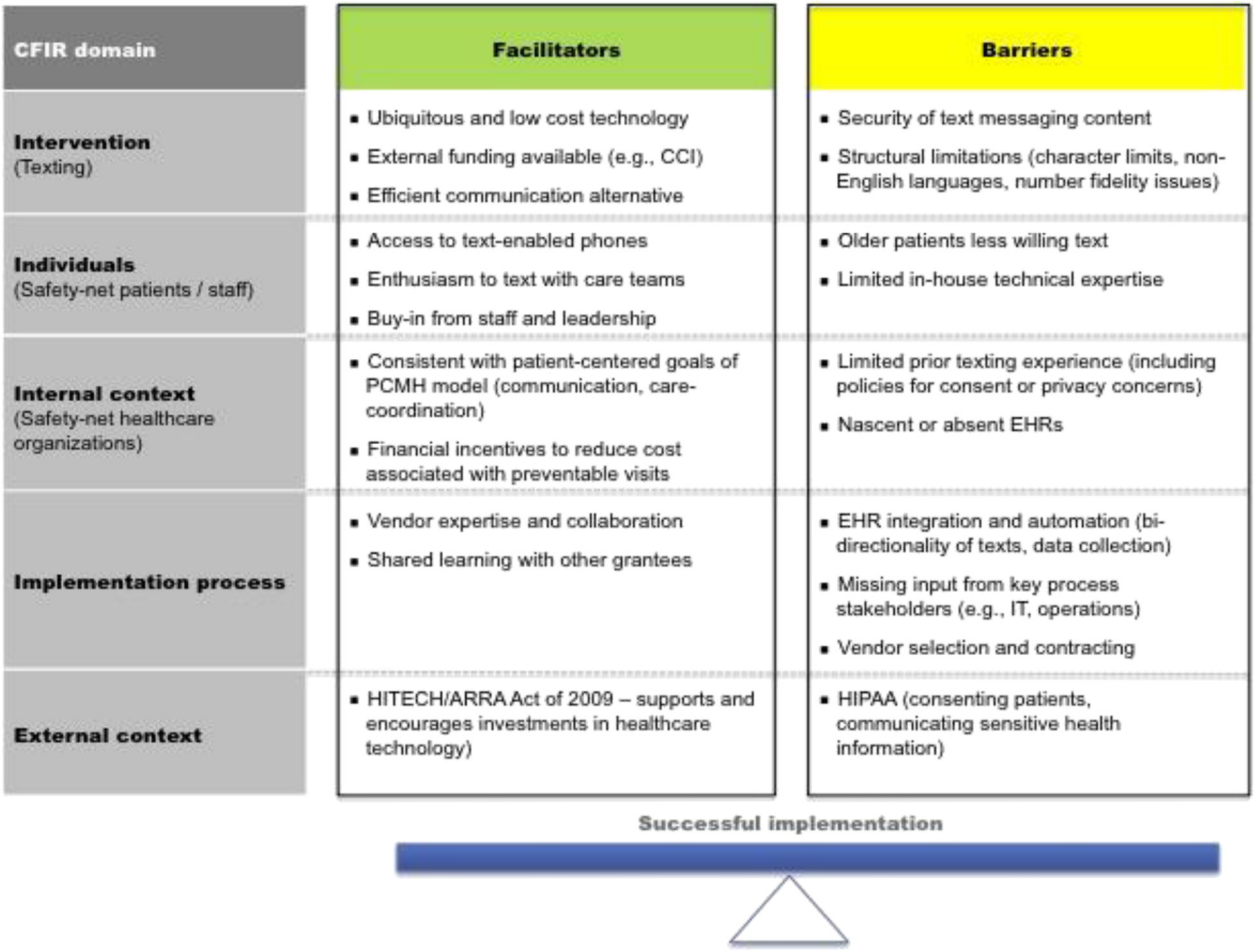
Facilitators and barriers of implementing pilot texting programs in the medical safety net^1^

We identified several facilitators and barriers to implementing patient texting programs, and categorized each facilitator and barrier within the CFIR domains (Fig. 2). Briefly, we identified several important *facilitators* within CFIR domains involving the “individuals” (perceived support from participating patients and providers) and the “internal setting” (alignment of communicating with patients through text with organizational emphasis on patient-centeredness). In contrast, several important *barriers* emerged within the CFIR domains of the “process” (data management and integration challenges) and the “outer setting” (confusing applications of privacy and security regulations to mobile health technology). The texting “intervention” itself was both a facilitator (affordable and ubiquitous technology) and a barrier (data security concerns of text message content for protected health information).

### Facilitator: Patient and staff engagement and support

All interviewed health systems felt that the majority of their patients were interested in texting, although two sites said that older patients were less interested. Participants also reported that most of their patients had cell phones and “used texting in their daily life.”

*“Patients actually have a preference for communicating by text. They [like] having better access and they like having answers sooner.” [Site F]*

Organizational enthusiasm for texting was reported to be equally strong among providers and senior management. Both sites A and C that experienced delays in launching their pilots also remained optimistic about their texting programs. Pilot texting programs were viewed as stepping-stones to other more robust applications of texting.

*“We presented it at our big clinician team meeting where we get all the providers from all of our clinics and we demonstrated the texting platform. Right away, providers see the benefit of that and so the questions start rolling in.” [Site F]*

### Facilitator: Perceived utility of text messages to accomplish health system goals

Participants reflected on the potential for texting to improve both care coordination and communication for patients outside of the clinic and between visits. Site E, with no prior texting experience, stated the following:

*“We obviously recognize that this [texting] is the natural evolution of patient care… So any way that we can - as their medical home - have methods of communication that we can be in contact with them, we view it as a major asset and tool.” [Site E]*

For example, the physician lead from Site C (achieved bidirectional texting capability and used an opt-out consent process) explained how the efficiency of high volume and asynchronous communication through texting improved their triage and workflows for hospital discharges. Specifically, patient responses to two simple “yes or no” questions (need for medication refills or a follow-up appointment) following discharge allowed the health system to quickly identify patients at increased readmission risk.

### Facilitator: Early identification of barriers and solutions through peer collaboration

Site teams benefited from real-time collaboration with other grantees early in the pilot process, creating an environment conducive to a “learning health system.” Important topics for this shared learning included how to work effectively with vendors and manage patient privacy and consent issues (Table 3). Pilot sites cited examples where the early identification of barriers encountered by other sites helped to improve their own implementation plans before launch. Site H had no prior texting experience and used an opt-in consent process, stating the following:

*“It’s nice to hear about what other people are doing to try and learn from them. The materials around different consent forms were very helpful for us in terms of adapting the one that we currently landed with.” [Site H]*

**Table 3:** Topics for shared learning in implementing patient texting in the medical safety net highlighted by pilot sites

Topics	Example quote(s)
Vendor selection	• “They [CCI] offered us vendors to look at too so I did my research on the different vendors and see what kind of innovations they have and platforms they have for texting.” [A]
Vendor contracts/payment	• “We don’t have to pay for each text message. Many of the other vendors actually charge for each text message and we were on the call this morning and they [another site] were saying that each text message is three-quarters of a cent.” [D]
Understanding HIPAA	• “I know one of the grants really had a huge HIPPA sort of concerns and put a hold on it.” [D] • “They pulled together a webinar on HIPAA. I would say that was a big plus for us.” [B] • “It might also be really helpful to either see, witness, or get connected with industries or organizations that are much more advanced maybe in the HIPAA area. So they’ve already figured out how to do it.” [F]
Designing consent forms	• “The consent piece was huge in terms of shared learning among grantees. [B] • “We both found it useful just to hear some of the other challenges that people were up against, particularly around the consenting piece.” [E]
Reassurance and support	• “I think it’s that reassurance that we’re not the only ones having some technical issues too in the project” [G] • “So having issues with their contractor …that’s interesting to see how they’re approaching it. I just found that useful.” [G]
Idea generation	• “I think learning that there is this program which we could potentially use to provide cellphones for our homeless population so they can participate. It has been really an endpoint learning for us.” [H] • “I’m not sure we would have come up with this idea had we not had some of the other observation tools through CCI.” [E] • “It [conference calls] has generated a lot of ideas for what we just liked to do with texting in the future, because they have a diabetes management platform that works within.” [C]

### Barrier: Electronic data integration

Half (four sites) of the pilot texting platforms were not sophisticated enough in design to allow for bi-directional communication in which patients could respond to provider messages (and providers to patients). Of the three sites with prior texting experience before the pilot, only one designed for bi-directionality.

Only three sites (Table 1) were able to integrate data from the texting platform with their existing EHR. Site B, with no prior texting experience, had an in-house programmer who performed most of the customization and integration, while the others two sites depended on vendors for this technical expertise. Site D, which had prior texting experience and an integrated platform, stated the following:

*“The cancellation that the patient is not [coming to their visit]– it’s connected to the EHR or EPM side of [this], so that automatically releases an appointment. Nobody has to do anything…which is gold. So that’s automated.” [Site D]*

The sites that were unable to integrate the texting platform with their EHR stated they had older EHR systems, were still in the midst of major overhauls and changes to their EHRs, or had interfacing roadblocks between the vendor platform and their EHR. Two sites tried to use the EHR itself to send texts to avoid integration issues but found the functionality of that texting option to be inferior to external vendor platforms. One was still using paper charts:

*“We’re actually on paper charts in most of clinics right now. So long term, actually the goal, we’re going to go on to [a new EHR system]. The goal is to have our texting platform interfaced with [our new EHR system].” [Site F]*

Overall, the lack of integration had important ramifications that limited data collection and created new labor-intensive tasks (e.g., tracking which patients consented or translating text responses into clinical decisions). Updating outdated phone numbers or documenting which phone numbers were not capable of receiving texts also became a cumbersome manual process for sites that had a separate EHR system and texting interface.

### Barrier: Protecting patient privacy and securing message content

No health system in our sample included patient-identifying health information in the message content. Pilot sites also faced many challenges around obtaining patient consent that significantly affected the robustness and speed of pilot rollouts (Table 4). This was driven in large part by their concerns of violating the US Health Information Portability and Accountability Act (HIPAA), which protects the confidentiality and security of healthcare information, including implications for procedures to obtain patient consent. Due to a consistent lack of clarity in applying HIPAA to texting, the default was *not* to include sensitive health information. Site D, which consented each individual patient, was still concerned about the legal consequences of patients who lost or did not password protect their phones, instructing patients instead to report blood pressure readings using a complicated coding system.

**Table 4:** Privacy and security factors affecting implementation of patient texting programs in the medical safety net

Theme	Example quote(s)
Risk-averse culture in safety net	• “In the safety net or especially in county facilities, we worry too much about all of the uncertainty. We tend to be very risk-averse.” [F] • “In terms of a starting point for doing texting, we thought it would be easier to start with that [opt-in consent], given our current privacy policies.” [H] • “I think opting in was an easier way of selling the campaign at that time.” [E] • “We chose to focus on self-management goals …then the texting conversations are not about typical HIPAA-sensitive items.” [F]
Ambiguity of HIPAA/privacy applications to texting	• “We got the green light to go ahead without a written consent…but then we were told that we can’t do that…it’s somewhat of a setback.” [H] • “We would love to be able to figure out how to communicate HIPAA-sensitive information going forward.” [F] • “We never felt like we got a straightforward answer as to whether we could just go with an opt-out plan, opt out system.” [E] • “In working with our privacy people, they said that we have to consent patients prior to initiating texting.” [H]
Concerns on security of text information	• “If it’s anything more confidential like regarding your test results and stuff, we will have to call them.” [A] • “There were some concerns about…the blood pressure information that we’re sending back and forth…there are different schools of thought…a lot more discussion to come in the future with all of this. [D]
No administrative precedent for texting policies/procedures	• “The technical side is done. We’re waiting on [administration] to basically approve this, because they don’t have a policy on texting yet and so it’s at the higher levels of the organization.” [C] • “We used it as an opportunity to update our patient consent… signing covers all of this electronic communication.” [B] • “We’re one of seven grantees in this CCI-funded initiative. I think we’re the only ones or one of the few that haven’t had a big hiccup with the consent process.” [B]
Opt-in consent process is labor-intensive and inefficient	• “Keeping track of who actually signed that consent…seems rather challenging.” [D] • “There are so few people who said they don’t want to get it [texts]. If someone doesn’t want to get it, it wouldn’t even be worth the discussion.” [B] • “We did a consent all with an opt-out option…with 100,00 patients and 400,000 encounters a year, we don’t have the luxury of opting [in] as we go.” • “They developed an auto opt out process to save time.” [B] • “When we had to convert to a written consent form, I think there is a lot less interest.” [H]

Similarly, six out of the eight sites chose to use a labor-intensive “opt-in” consent process that involves having the staff individually obtain informed consent from each patient through a written agreement (Table 1). This was often done to avoid the administrative hurdles of changing existing organizational privacy policies. A simpler “opt-out” approach assumes up-front patient consent until an individual responds “no” after receiving an initial text. The two remaining sites (B and C) took a hybrid approach to an opt-out process by incorporating texting into an up-front universal consent process that included other forms of communication like telephone or email. However, both of these sites had bi-directional communicating capability that could allow patients to state they no longer wanted to receive texts. Using this hybrid approach, Site B sent 5,000 messages per week and said 10 % of patients opted-out of text messaging, as compared to a site using an opt-in consent that sent a few hundred texts per week and said 40 % of patients chose to not receive texts.

## Discussion

In this qualitative assessment of the real-world implementation of clinical texting programs to communicate with patients in the California safety net, application of the CFIR framework enhanced our understanding of the issues affecting the uptake of texting interventions, yielding actionable implementation lessons. We found that most of the studied organizations encountered common implementation facilitators and barriers that affected the adoption of texting technology. Texting pilot programs addressed a variety of patient needs that were in line with internal organizational priorities and were not met with overt resistance from providers or patients. However, concern and confusion around the application of government privacy and security regulations led to cumbersome consent procedures and less robust clinical platforms that did not include sensitive health information. Difficulty in integrating texting platforms with electronic health records also posed a significant barrier to realizing the potential benefits of automating and archiving texting conversations and responses.

Text messaging falls under a larger umbrella of digital health interventions termed “mHealth,” or mobile health. The World Health Organization (WHO) defines mHealth as the use of mobile and wireless technologies, specifically a mobile phone’s core utility of voice and short messaging service (SMS) as well as more complex functionalities and applications, to support the achievement of health objectives [33]. In a global survey of implementation barriers, the WHO found that competing health system priorities were the largest barrier to mHealth adoption and was thought to be due primarily to the lack of demonstrated efficacy of mHealth interventions relative to more established health interventions. A review by Becker et. al. suggests this insufficient evidence base is limiting the scaling up mHealth initiatives beyond the majority of smaller pilot initiatives that are reported to date [21]. Tomlinson et. al. calls specifically for major governmental and private investment to build a more robust, standardized, and interoperable health information platform with open architecture that can then facilitate the larger and more complex mHealth research initiatives that are needed to further demonstrate efficacy [27]. The WHO similarly concludes in their survey that policy and legal challenges, such as health information security, patient confidentiality, and interoperability, are also important implementation barriers for scaling up this technology.

Our study findings are largely consistent with the published literature on mobile texting between health systems and patients, and suggest the potential uses and challenges of implementing texting are also present in safety net settings. The eight pilot sites in our study used texting for a range of different clinical and administrative functions, which is consistent with the variety seen in the published literature in both non-safety-net [11–19] and safety net [25, 26, 28, 29] settings. The suggestion from prior studies [8–10] that underserved patients are willing and able to text with their providers is also consistent with the perceptions of health systems regarding their patients’ willingness to text in our study. However, texting intervention studies conducted to date have been mostly limited by small sample sizes [34, 35]. Our study adds to the literature by encompassing multiple pilots with a focus on examining the implementation of texting itself and also exploring provider perspectives, both of which are less well-studied [34]. A health promotion pilot study by Albright et. al. that was conducted in rural undeserved areas found that no patient had concerns about text messaging and concluded that patients generally favored receiving informative, clear and actionable texts but differed in their desire for interactivity of texting communications, or bi-directionality [25]. Contrary to these patient perspectives, we find that the safety net organizations we studied do have real concerns about implementing texting, which may explain why the majority of pilot intervention studies to date have been small in scale. We also found that many of the same challenges discussed in the existing literature around the use of mobile texting technologies, such as health privacy, data security, and electronic data integration, are also affecting safety-net health systems. However, we believe that these challenges, when combined with the added limitations (financial, infrastructural, or otherwise) affecting resource-limited health systems, may explain the slower adoption of texting technologies.

Legal considerations around protecting both patient privacy and health information security emerged as important barriers to implementing texting programs in our study. The US Health Information Technology for Economic and Clinical Health Act of 2009 widened the scope and enforcement of HIPAA protections in the context of electronic protected health information (or e-PHI). In addition, at least five different US government agencies play partial roles in monitoring healthcare mobile device use, and no single federal agency has been given primary authority to enact these protections [36, 37]. Our results demonstrate the resulting confusion and uncertainty in interpreting privacy rules with regard to texting, and suggest that clear legal and policy guidelines would facilitate implementation of texting programs in healthcare systems. These barriers are not new and have been raised prior to our study in the mHealth literature [38].

Uncertainty in interpreting HIPAA regulations also applied to consenting procedures for obtaining permission from patients to communicate with them through texts. In 2012, the US Federal Communications Commission (FCC) rejected class-action lawsuits regarding the use of first-contact “opt-out” confirmation text messages with non-health consumers, but we are not aware of any such legal precedents for healthcare patients [39]. In 2013, the FCC also updated the Telephone Consumer Protection Act to require “express written consent” for commercially sent text messages from automatic dialing systems, however exemptions were made for “healthcare messages” regulated under HIPAA. “Healthcare messages” were loosely defined in the legislation and the exemptions are ambiguous in interpretation [40]. The health systems in our study had differing approaches to consenting patients. “Opt-in” consenting processes (the majority of the health systems in our study) are more labor-intensive and may slow the rollout and rapid scaling of text-messaging programs. Legal and regulatory clarity is needed around best practices to obtain patient consent for texting communications. Until then, an interim solution may be to use the hybrid approach of higher-volume implementers in our study of including “text-messaging” in universal consenting process already in place to communicate with patients through phone, postal mail, electronic mail, or internet-based portals. However, an additional challenge in consenting patients that we were not able to explore in this study involves communicating the complexity of risk inherent in mHealth technology and respecting the variability in risk profiles and preferences of individual patients [41].

HIPAA not only added complexity to the consent process, which may have limited the scale of some texting programs, but also limited the content of the text messages. Boisvert et. al. conclude texting is not designed to deliver protected and sensitive information but should instead be used as an adjunct to improve patient care [42]. Because of the concern over ensuring the appropriate security safeguards on patient mobile devices, all pilot sites in our sample elected to withhold any patient-identifying health information in their communications. Similarly, none of the interventions of prior texting studies in disadvantaged populations that we identified sent sensitive health information [25, 26, 28, 29]. Excluding protected health information limits the utility of text messaging for supporting disease self-management or tailored recommendations. We believe this is a missed opportunity in current texting programs for providers to use patient-generated health data and deliver customizable care plans [38], although one prior study suggests that patient attitudes towards mHealth privacy and security may be variable from person-to-person and context-dependent [43]. Health systems are unable to ensure the security of text message content given that the adequacy of safeguards often lies outside the HIPAA-regulated clinical care setting (e.g., password protection by patients or data encryption sophistication by service providers) [42]. Commercial interfaces for secure messaging are emerging, but the use of these applications may be less feasible in resource-limited health settings due to cost barriers and smartphone requirements.

Another key advantage of text messaging is the ability to archive data within such a platform. However, the lack of integration with electronic health records precluded this potential benefit of texting in our sample. A survey of 194 community health centers throughout the state of California found that while 80 % used an EHR, nearly 50 % were not interoperable (able to receive outside data into the EHR) [44]. While this may be because of the lack of electronic health record infrastructure in safety net settings [4], the challenge of integrating independent platforms with electronic health records extends across all US health care systems [45]. If electronic health record vendors can add/enhance options to text patients directly from the EHR or permit open application programming interfaces (APIs) that allow for the development of better software-to-software communication, this has the potential to further enhance the utility of texting and enable larger research studies. Such integration would also help in documenting e-PHI communications with patients for legal purposes [36].

To our knowledge, our analysis is the first multi-site study of the implementation of texting in healthcare and focuses on the provider experience whereas most published pilots focus on patient experience and outcomes. While our sample of eight safety-net health systems likely differs from private and for-profit health systems, our findings highlight general challenges for all healthcare systems as well as specific potential barriers for systems serving predominantly diverse and underserved patient populations. While the number of interviews in our study is modest and precludes drawing definitive conclusions, we believe the facilitators and barriers we uncovered are relevant across healthcare settings. Moving forward, future implementation work and research should rigorously investigate how different texting platform and intervention designs affect efficacy, as well as explore issues that may affect sustainability and the scalability of these interventions.

Some important limitations to consider include that all of our participating sites were located in California, and hence legal or other implementation challenges encountered by our sites may be distinct from those in other settings. We also did not directly interview patients which limited are ability to explore patient-level facilitators, such as enhancing healthcare access, or barriers, such as language (literacy, English proficiency), cost (of texting to patients), or privacy concerns. We hope to explore these and other patient-level factors once these pilots expand/extend, at which point we can also study whether or not the implementation efforts in this analysis were sustained. The award of external funding, albeit small in amount, for all pilot sites in our study also makes it harder to comment on health system cost barriers to implementation. Our interviews also did not explore the challenges related to the future costs of sustaining, evaluating or improving pilot interventions. With respect to our interviewing methodology, our use of multiple interviewers may have resulted in variations in our data collection. Also, given that four out of the eight site interviews had multiple participants often with varying leadership and organizational roles, we also acknowledge that responses may have been affected by unknown power dynamics or the group compositions themselves. Determining organizational roles for each of the interviewed respondents required post-hoc follow-up since we could only partially collect this information using the telephone transcripts as not all participants were asked or provided this information during the interview. We were also unable to accurately match transcribed interview responses to a particular individual using the interview transcripts, and therefore were not able to reflect in detail on how participant views may have differed according to organizational roles or group interview dynamics.

## Conclusion

Texting is an affordable, ubiquitous and emerging technology that may help health systems to improve the quality of health services. Support from patients, providers and leadership facilitated the implementation of texting programs in the safety net, including the participation in a peer-based “learning health system” model. In contrast, patient privacy and health information security concerns, as well as lack of EHR integration, impeded progress. Improving the robustness of clinical texting applications over the longer term will require more infrastructure investment in EHR systems, open APIs, and clarification/simplification of federal health regulations as they relate to mobile health technology. To promote faster adoption of texting in the shorter term, health systems in the safety net should consider including texting into universal consent procedures for electronic communications, and partner closely with vendors, legal advisors and peer health systems who have experience with texting. We believe these early results from safety net systems can inform a broad range of health systems interested in implementing texting programs.

## Additional file

Additional file 1:
**Texting Interview Questions.** (DOCX 16 kb)

## Abbreviations

API: application programming interface
CCI: Center for Care Innovations
CFIR: Consolidated Framework for Implementation Research
FCC: Federal Communications Commission
EHR: electronic health record
e-PHI: electronic protected health information
HIPAA: health insurance portability and accountability act
IT: information technology
PCHM: patient-centered medical home
PHoENIX: Public Healthcare system Evidence Network and Innovation eXchange
US: United States

## References

[1] Bates DW, Bitton A (2010). The future of health information technology in the patient-centered medical home. Health Aff.

[2] Sheikh A, Sood HS, Bates DW (2015). Leveraging health information technology to achieve the “triple aim” of healthcare reform. Journal of the American Medical Informatics Association : JAMIA.

[3] Institute of Medicine. America’s Healthcare Safety Net: Intact but Endangered. 2000.

[4] Jha AK, DesRoches CM, Shields AE, Miralles PD, Zheng J, Rosenbaum S (2009). Evidence of an emerging digital divide among hospitals that care for the poor. Health Aff.

[5] Katz MH (2010). Future of the safety net under health reform. JAMA.

[6] Pew Research Center. Mobile Technology Fact Sheet. http://www.pewinternet.org/fact-sheets/mobile-technology-fact-sheet/. Last accessed 01/27/2015.

[7] Pew Research Center. Trends and demographic differences in home broadband adoption. http://www.pewinternet.org/2013/08/26/home-broadband-2013/. Last accessed 01/27/2015.

[8] Schickedanz A, Huang D, Lopez A, Cheung E, Lyles CR, Bodenheimer T (2013). Access, interest, and attitudes toward electronic communication for health care among patients in the medical safety net. J Gen Intern Med.

[9] McInnes DK, Sawh L, Petrakis BA, Rao S, Shimada SL, Eyrich-Garg KM (2014). The potential for health-related uses of mobile phones and internet with homeless veterans: results from a multisite survey. Telemedicine journal and e-health : the official journal of the American Telemedicine Association.

[10] Asgary R, Sckell B, Alcabes A, Naderi R, Adongo P, Ogedegbe G. Perceptions, Attitudes, and Experience Regarding mHealth Among Homeless Persons in New York City Shelters. Journal of health communication. 2015:1-8. doi:10.1080/10810730.2015.1033117. 10.1080/10810730.2015.1033117 PMC4654657 26313765

[11] Gurol-Urganci I, de Jongh T, Vodopivec-Jamsek V, Atun R, Car J (2013). Mobile phone messaging reminders for attendance at healthcare appointments. The Cochrane database of systematic reviews.

[12] Alvarez-Jimenez M, Alcazar-Corcoles MA, Gonzalez-Blanch C, Bendall S, McGorry PD, Gleeson JF (2014). Online, social media and mobile technologies for psychosis treatment: a systematic review on novel user-led interventions. Schizophr Res.

[13] Buchholz SW, Wilbur J, Ingram D, Fogg L (2013). Physical activity text messaging interventions in adults: a systematic review. Worldviews on evidence-based nursing / Sigma Theta Tau International, Honor Society of Nursing.

[14] Head KJ, Noar SM, Iannarino NT, Grant HN (2013). Efficacy of text messaging-based interventions for health promotion: a meta-analysis. Soc Sci Med.

[15] Kong G, Ells DM, Camenga DR, Krishnan-Sarin S (2014). Text messaging-based smoking cessation intervention: a narrative review. Addict Behav.

[16] Shaw R, Bosworth H (2012). Short message service (SMS) text messaging as an intervention medium for weight loss: A literature review. Health Informatics J.

[17] Vodopivec-Jamsek V, de Jongh T, Gurol-Urganci I, Atun R, Car J (2012). Mobile phone messaging for preventive health care. The Cochrane database of systematic reviews.

[18] Whittaker R, McRobbie H, Bullen C, Borland R, Rodgers A, Gu Y (2012). Mobile phone-based interventions for smoking cessation. The Cochrane database of systematic reviews.

[19] Saffari M, Ghanizadeh G, Koenig HG (2014). Health education via mobile text messaging for glycemic control in adults with type 2 diabetes: a systematic review and meta-analysis. Prim Care Diabetes.

[20] Nundy S, Dick JJ, Chou CH, Nocon RS, Chin MH, Peek ME (2014). Mobile phone diabetes project led to improved glycemic control and net savings for Chicago plan participants. Health Aff.

[21] Becker S, Miron-Shatz T, Schumacher N, Krocza J, Diamantidis C, Albrecht UV (2014). mHealth 2.0: Experiences, Possibilities, and Perspectives. JMIR mHealth and uHealth.

[22] Nhavoto JA, Gronlund A (2014). Mobile technologies and geographic information systems to improve health care systems: a literature review. JMIR mHealth and uHealth.

[23] Qiang CZ, Yamamichi M, Hausman V, Altman D (2011). Mobile applications for the health sector.

[24] Yeager VA, Menachemi N (2011). Text messaging in health care: a systematic review of impact studies. Advances in health care management.

[25] Albright K, Krantz MJ, Backlund Jarquin P, DeAlleaume L, Coronel-Mockler S, Estacio RO. Health Promotion Text Messaging Preferences and Acceptability Among the Medically Underserved. Health promotion practice. 2015. doi:10.1177/1524839914566850. 10.1177/1524839914566850 25586133

[26] McInnes DK, Petrakis BA, Gifford AL, Rao SR, Houston TK, Asch SM (2014). Retaining homeless veterans in outpatient care: a pilot study of mobile phone text message appointment reminders. Am J Public Health.

[27] Tomlinson M, Rotheram-Borus MJ, Swartz L, Tsai AC (2013). Scaling up mHealth: where is the evidence?. PLoS Med.

[28] Dick JJ, Nundy S, Solomon MC, Bishop KN, Chin MH, Peek ME (2011). Feasibility and usability of a text message-based program for diabetes self-management in an urban African-American population. J Diabetes Sci Technol.

[29] Nundy S, Razi RR, Dick JJ, Smith B, Mayo A, O’Connor A (2013). A text messaging intervention to improve heart failure self-management after hospital discharge in a largely African-American population: before-after study. J Med Internet Res.

[30] George S, Garth B, Fish A, Baker R (2013). Factors shaping effective utilization of health information technology in urban safety-net clinics. Health Informatics J.

[31] Center for Care Innovations. “Texting For Better Care.” http://www.careinnovations.org/programs-grants/texting/.

[32] Damschroder LJ, Hagedorn HJ (2011). A guiding framework and approach for implementation research in substance use disorders treatment. Psychology of addictive behaviors : journal of the Society of Psychologists in Addictive Behaviors.

[33] World Health Organization. Second Global Survey on eHealth (Global Observatory for eHealth) Geneva: World Health Organization; 2011. [2015-02-16]. webcite mHealth: New horizons for health through mobile technologies. http://apps.who.int/iris/bitstream/10665/44607/1/9789241564250_eng.pdf.

[34] Jones KR, Lekhak N, Kaewluang N (2014). Using mobile phones and short message service to deliver self-management interventions for chronic conditions: a meta-review. Worldviews on evidence-based nursing / Sigma Theta Tau International, Honor Society of Nursing.

[35] Hall AK, Cole-Lewis H, Bernhardt JM (2015). Mobile text messaging for health: a systematic review of reviews. Annu Rev Public Health.

[36] Washington L (2012). Managing Health Information in Mobile Devices. J AHIMA.

[37] Hall JL, McGraw D (2014). For telehealth to succeed, privacy and security risks must be identified and addressed. Health Aff.

[38] Petersen C, DeMuro P (2015). Legal and regulatory considerations associated with use of patient-generated health data from social media and mobile health (mHealth) devices. Applied clinical informatics.

[39] Federal Communications Commission. Declaratory Ruling re: SoundBite TCPA Petition. http://www.fcc.gov/document/declaratory-ruling-re-soundbite-tcpa-petition. Last accessed 01/29/2015.

[40] Roth, MS, Pfister HR, Karl AO. “Health Care-Related Calls: Ambiguity at the Intersection of HIPAA and TCPA.” Bloomberg PNA Privacy and Security Law Report. 13 PVLR 1756, 10/13/2014. http://www.manatt.com/Articles/_Health_Care_-Related_Calls__Ambiguity_at_the_Intersection_of_HIPAA_and_TCPA.aspx.

[41] Carter A, Liddle J, Hall W, Chenery H (2015). Mobile Phones in Research and Treatment: Ethical Guidelines and Future Directions. JMIR mHealth and uHealth.

[42] Boisvert S (2012). An enterprise look at mHealth. Journal of healthcare risk management : the journal of the American Society for Healthcare Risk Management.

[43] Atienza AA, Zarcadoolas C, Vaughon W, Hughes P, Patel V, Chou WY (2015). Consumer Attitudes and Perceptions on mHealth Privacy and Security: Findings From a Mixed-Methods Study. J Health Commun.

[44] Kim KK, Rudin RS, Wilson MD (2015). Health information technology adoption in California community health centers. Am J Manag Care.

[45] Weber GM, Mandl KD, Kohane IS (2014). Finding the missing link for big biomedical data. JAMA.

