# Supplementary material for: Qualitative analysis of programmatic initiatives to text patients with mobile devices in resource-limited health systems
Source: BMC Med Inform Decis Mak. 2016 Feb 6;16:16. doi: 10.1186/s12911-016-0258-7 (PMC4744448; doi:10.1186/s12911-016-0258-7)
Supplement: Additional file 1: — Texting Interview Questions. (DOCX 16 kb) [file 12911_2016_258_MOESM1_ESM.docx]

**APPENDIX**

**Texting Interview Questions**

1. Why did you want to incorporate texting into clinical care at your location?
   1. Would you have done this without the funding from CCI? or
   2. Was the CCI funding the driver of the texting program?
2. Was texting the chicken or the egg?
   1. What was driving adoption – texting availability or clinical need?
3. What are your primary goals with this texting program?
   1. Probe: Are you primarily trying to improve the quality of care, or the efficiency of care with this service?
   2. Is there a specific concern about patient satisfaction you’d want to improve?
4. Is texting offering a new service to patients?
5. Will texting require a change in your workflow?
6. How did you decide to focus on this particular content for the pilot?
   1. Probe: from within the organization, or externally
   2. Top-down or bottom up?
   3. Patient requests?
   4. Probe: evidence?
7. How is/was providing information related to the pilot currently handled at your location?
8. Compared to your current procedures for managing providing information about access to health insurance, what do you think are the advantage(s) of using text messaging?*
9. What do you see as the biggest advantages and disadvantages of working with your vendor [insert here]?
   1. Do you feel like there is enough flexibility in the texting service to make it work best at your facility?
10. Do you have plans to integrate texting into EHR?
11. What kinds of meetings and other communications do you engage in that are important for your texting program to meet its goals?
12. What measures are you using to see if texting works?
13. Do you have baseline data?
14. Tell us about your HIPAA/ consent process
15. How has that affected your implementation?
16. Have you encountered any (other) technical problems?
17. Have you encountered any other implementation and/or operational challenges beyond what you just talked about?
    1. If piloting/implementing already ask the following:
       1. Accurate phone numbers from participants?
       2. Appropriate roles appointed for implementing texting in your site?
       3. Buy in achieved at your site?
       4. Adopt technology changes: Internally? Externally?
       5. Measure outcomes?
       6. Integrate into care?
       7. Other?
18. How do you think the program is doing in terms of meeting its goals?
    1. Has anything made it difficult to implement the program?
19. Do you have sufficient resources to support your texting program
    1. What resources are needed?
    2. During grant period
    3. After grant period
20. Are there people at your facility who have been particularly important in helping to get the texting program funded and implemented?
    1. Roles as internal or external to your day to day work?
21. (If already starting to pilot texting): How receptive have patients been to receiving texts?
    1. Could you identify factors that helped get them to adopt texting?
       1. Walk us through it
22. Have you been in contact with other CCI grantees?
23. Has this helped with your implementation in any way?
